# Assessment of Liver Involvement in Patients With Type 2 Diabetes Mellitus in Basrah City, Iraq, Using FibroScan and Correlation With Risk Factors

**DOI:** 10.7759/cureus.65089

**Published:** 2024-07-22

**Authors:** Muntadher A Abdullah, Fatima J Kadhum, Sajjad S Issa

**Affiliations:** 1 College of Medicine, University of Basrah, Basrah Gastroenterology and Hepatology Hospital, Basrah, IRQ; 2 Medicine, Al-Fayhaa General Hospital, Basrah, IRQ; 3 Family Medicine, College of Nursing, University of Basra, Basrah, IRQ

**Keywords:** transient elastography, risk factors, nonalcoholic fatty liver disease, fibroscan, diabetes mellitus

## Abstract

Background

Non-alcoholic fatty liver disease (NAFLD) is a spectrum of liver diseases caused by the accumulation of fat in the liver, which can progress to fibrosis, cirrhosis, and primary liver cancer. Insulin resistance is a causative factor in the development of NAFLD. FibroScan, or transient elastography, is a noninvasive imaging technique for evaluating liver disease.

Aim

To use FibroScan for evaluating liver involvement in type 2 diabetes mellitus (T2DM) patients with some associated risk factors.

Materials and methods

A cross-sectional prospective study was conducted from February to August 2023 in the outpatient clinic of Basrah Gastroenterology and Hepatology Hospital. Data collection included demographic data, past medical history, and biochemical tests including fasting blood sugar (FBS), aspartate aminotransferase (AST), alanine aminotransferase (ALT), and lipid profile (consisting of low-density lipoprotein (LDL), high-density lipoprotein (HDL), serum triglycerides, and total cholesterol), then, patients underwent FibroScan examination.

Results

The study included 50 patients with T2DM, of whom 23 (46%) were male and 27 (54%) were female. The mean age of the studied population was 47.72 ± 8.31 years, with a range of 28-64 years. The mean BMI was 28.44 ± 4.24, with most patients being either overweight or obese. The fibrosis score was 4.74 ± 1.02 kPa (stage 0), while the mean steatosis score was 282.88 ± 44.99 (grade III). Diastolic blood pressure (BP), serum ALT, and serum HDL level were the variables that showed statistically significant differences when compared according to the stages of steatosis measured by FibroScan, with p-values of 0.016, 0.048, and 0.028, respectively.

Conclusion

Some risk factors associated with diabetes, such as dyslipidemia, liver enzymes, and BP, are highly associated with the development of steatosis rather than fibrosis.

## Introduction

Non-alcoholic fatty liver disease (NAFLD) is a type of liver disease in which the liver accumulates fat without being overly dependent on alcohol. The severity of NAFLD can range from simple fatty liver with inflammation to damage to the hepatocytes, and in the most severe cases, it can develop into cirrhosis and hepatocellular cancer [1]. NAFLD is one of the most common forms of chronic liver disease worldwide [2].

Type 2 diabetes mellitus (T2DM) is linked to a number of chronic liver disorders in addition to atherosclerosis, cardiovascular disease, chronic kidney disease, and cancer. Patients with T2DM may face a wide range of complications, and their life expectancy can be reduced by an average of 10 years. Similar to chronic viral hepatitis, long-term nonalcoholic hepatitis can lead to liver fibrosis, cirrhosis, and even end-stage liver diseases [3].

Individuals with T2DM are more likely to develop hepatic fibrosis/cirrhosis, non-alcoholic steatohepatitis (NASH), and NAFLD. A major contributing factor to the pathophysiology of NAFLD is insulin resistance. Furthermore, the mortality rate for NAFLD patients with DM is three times higher than that of NAFLD patients without DM [4].

Insulin resistance causes hyperinsulinemia, which increases glycolysis and decreases apolipoprotein B-100, subsequently reducing VLDL export and increasing intra-hepatocytic fatty acids [5].

Fibrosis staging is crucial for all NAFLD patients to identify those with advanced hepatic fibrosis at risk for liver-related complications, hepatocellular dysfunction, and portal hypertension brought on by progressive hepatic fibrosis [5]. Liver diseases were the tenth most common cause of death worldwide [6]. To forecast the prognosis for NAFLD, surveillance and effective treatment should be undertaken to prevent progression to advanced liver diseases [7].

NAFLD can affect people of different ages, genders, and races. The main risk factors for NAFLD are obesity, metabolic syndrome, diabetes mellitus (DM), and hyperlipidemia, with the latter presenting the most significant risk [8].

Liver biopsy is the gold standard for diagnosing NAFLD but is sometimes associated with complications such as bleeding, bile leakage, infection, and other potentially fatal complications. FibroScan, a noninvasive method for determining liver stiffness and diagnosing fibrosis, was developed as a result of the limited diagnostic accuracy of MRI, CT, and ultrasound imaging for NAFLD [9-10].

The prevalence of and factors related to the increased risk of NAFLD in diabetic patients in Basrah, southern Iraq, remain unstudied to date. Data regarding the profile of liver fibrosis in the population are also unknown.

Diabetes and NAFLD are clearly related; recent research has demonstrated that both the onset of diabetes and NAFLD are predictive of one another, and that each condition acts as a catalyst for the other's progression, although may play a role in this relationship, it is also likely that diabetes interacts with NAFLD through distinct pathogenic pathways [11]. In this regard, future challenges for researchers and clinicians will involve raising awareness of the connection between diabetes and NAFLD and identifying the pathogenic factors that underlie both conditions, as well as developing noninvasive, cost-effective methods of screening for and diagnosing NAFLD progression in at-risk individuals and developing successful interventions for NAFLD in diabetes to prevent both systemic and hepatic aftereffects. Recent papers addressing diabetes pathogenesis have addressed in great detail the metabolic abnormalities of insulin resistance and hyperinsulinemia in T2DM, with a relative insulin deficit causing changes to lipid and protein catabolism as well as hyperglycemia [12].

Skeletal muscle and adipose tissue exhibit exceptional sensitivity to insulin, but post-receptor signaling pathways and specific cell surface receptors play a major role in classical insulin signaling. Hepatic insulin resistance and T2DM are also associated with these conditions. The liver dynamically controls glucose flow and metabolism, and as a result, glycemia through insulin-dependent mechanisms [13].

The pathophysiology of diabetes has been better understood in recent years, particularly with regard to the function of neuronal and endocrine transmission between the liver and brain. Along with chronic liver diseases of any etiology such as hemochromatosis, alcoholic liver disease, chronic viral hepatitis, and cirrhosis from any source, the prevalence of T2DM is also higher in NAFLD [14]. Increased insulin resistance in skeletal muscle, hepatic, and adipose tissues is linked to dysglycemia in chronic liver illness. Compensatory hyperinsulinemia and subsequent pancreatic endocrine (β-cell) dysfunction are also connected with dysglycemia [15].

As liver disease progresses, decreased glucose metabolism becomes increasingly significant; in individuals with cirrhosis, up to 96% have impaired glucose tolerance [16]. Hepatogenous diabetes, estimated to affect 30-60% of patients with cirrhosis, has a different clinical course from classical T2DM in that it is more often linked to complications resulting from cirrhosis than from the microangiopathic complications characteristic of diabetes [15].

Non-alcoholic liver disease prevalence

According to estimates, over 30% of Americans and over 25% of Asians suffer from NAFLD, making it the most prevalent liver disease globally [17]. Because a liver biopsy is necessary for a conclusive diagnosis, it is challenging to quantify the prevalence of NASH in the general adult population. However, it is frequently estimated to be between 3 and 5% in the US, including in recent US position guidelines [18].

It is undeniable that the incidence of NASH in various research cohorts, which show variability in NASH risk factors, differs substantially and is frequently noticeably higher than 3-5%. In one recent prospective study, for instance, 46% of 400 US military personnel and their families, with a mean age of 55 years, had evidence of steatosis on ultrasound, and 30% of these ultrasound-positive patients had NASH at biopsy, meaning that 12% of the study cohort as a whole had NASH [19].

During the study period, there was no rise in the prevalence of any other indication for liver transplantation. Furthermore, because the alterations associated with fatty liver may become less obvious on biopsy as fibrosis grows, these numbers are likely to underestimate the incidence of NASH as a reason for liver transplant. Additionally, they disregard the possible influence that NASH has on the other recognized chronic liver disorders [19].

Focusing on the results of liver biopsies to understand the natural history of liver morbidity in NAFLD in the general population, the presence of NASH was linked to a 17.5% risk of liver-related mortality over approximately 20 years of follow-up in a research series of 131 subjects [20]. This makes understanding the burden of NASH in the NAFLD population crucial. Hepatic steatosis alone is thought to be relatively benign from a liver perspective, with a 0-3% liver-related mortality rate over 10-20 years.

Among patients with NAFLD, the prevalence of peripheral vascular, coronary, and cerebrovascular diseases was noticeably greater. This result was unaffected by conventional macrovascular risk factors; the duration of diabetes; the degree of glycemic control; the use of antihypertensive, antidiabetic, lipid-lowering, or antiplatelet drugs; or elements of the metabolic syndrome. Furthermore, in patients with T2DM who were also known to have coronary artery disease, NAFLD was linked to decreased myocardial perfusion as determined by magnetic resonance spectroscopy. It was discovered that this was unrelated to visceral fat mass, conventional risk factors, and insulin sensitivity (measured by a euglycemic hyperinsulinemic clamp) [21].

Furthermore, vascular events (such as nonfatal myocardial infarction, nonfatal stroke, and vascular death) and vascular-related death were linked to a history of diabetes and hypercholesterolemia in a cohort of 247 Child-Pugh class A patients with biopsy-confirmed NAFLD with advanced fibrosis or cirrhosis. In this group, the adjusted odds ratio for both vascular events and deaths when diabetes was present was 10.43 (95% CI, 1.26-82.53; P=0.03). This increase in risk is greater by a factor of around 5 compared to the estimated risk of vascular disease that diabetes carries in the general population [22].

Thus, the aim of this study was to use FibroScan to evaluate liver involvement in T2DM patients and assess associated risk factors for this involvement.

Justification of the study

Liver involvement in diabetic patients is considered a challenging subject because such involvement might develop into fatty changes and associated complications; as such, the study of such subjects is vital.

## Materials and methods

A cross-sectional analytical study was conducted to assess liver disease among diabetic patients who attended the outpatient clinic of Basrah Gastroenterology and Hepatology Hospital from February to August 2023. A convenient sample of 50 patients was selected. All participants were diabetic patients (T2DM as per American Diabetes Association (ADA) criteria) treated with insulin, oral antidiabetic drugs, or a combination of both, aged 27 to 64 years old.

Patients with a history of alcohol intake, known cases of hepatitis B or C, ascites, morbid obesity, gestational DM, other chronic liver diseases (such as autoimmune hepatitis, hemochromatosis, Wilson's disease, primary biliary cirrhosis, drug-induced hepatitis), or T1DM, and those who refused to participate, were excluded from the study.

Medical history and demographic data were collected from patients who attended the outpatient clinic of Basrah Gastroenterology and Hepatology Hospital after meeting the inclusion and exclusion criteria and consenting to participate in the study. Fasting venous blood samples were drawn, and measurements of aspartate transaminase (AST), alanine transaminase (ALT), lipid profile (consisting of low-density lipoprotein (LDL), high-density lipoprotein (HDL), serum triglycerides, and total cholesterol), and fasting blood sugar were conducted. Subsequently, the selected patients underwent a FibroScan examination. In this procedure, a tiny transducer on the end of an ultrasonic probe emits a 50-MHz wave into the liver. The probe also incorporates a transducer that can measure the velocity of the shear wave at its tip. Essentially, the technique calculates liver stiffness measurement (LSM), expressed in kilopascals (kPa), from the velocity of the sound wave that passes through the liver. This measurement correlates well with the level of liver fibrosis in various liver disorders, including NAFLD [5]. The instrument used in the study was an Echosens FibroScan 530 COMPACT (2018), located at 30 Place d’Italie, 75013 Paris, France. For a test to be considered successful, 10 valid FibroScan readings are required, according to the manufacturer’s recommendations and results from previous research. A success rate of over 90% was achieved for every patient participating in the trial.

Based on cutoffs for fibrosis scores for NAFLD/NASH and also based on the official physician information sheet provided along with the FibroScan machine for the same category of patients, the fibrosis and steatosis scores of the population in this study were categorized into the following: FIBROSIS: F0 0-5.7 kPa, F1 5.8-6.7 kPa, F2 6.8-7.7 kPa, F3 7.8-11.8 kPa, F4 > 11.8 kPa; STEATOSIS: S0 < 240 dB/m, S1 = 240-265 dB/m, S2 = 265-295 dB/m, S3 >= 295 dB/m [23].

The normal range for serum ALT at the Gastroenterology and Hepatology Hospital is 12-78 U/L. Because determining a cutoff value for serum ALT is challenging and most researchers still use 30 U/L as the upper cutoff value, we adopted this value for the upper limit of serum ALT in this study [24].

The cut-off values for a normal lipid profile were as follows [25]: total serum cholesterol less than 200 mg/dl, LDL less than 100 mg/dl, fasting serum triglycerides less than 150 mg/dl, and HDL cholesterol greater than 60 mg/dl. Blood pressure (BP) classification was according to the ADA Clinical Practice Recommendation 2023 (ADA 2023), with BP ≥ 130/80 mmHg considered hypertensive in diabetic patients.

Statistical analysis

Statistical analysis was conducted using the SPSS version 26 (IBM Corp., Armonk, NY, USA) The Shapiro-Wilk and Kolmogorov-Smirnov tests were used to detect the normality of quantitative data distribution. Quantitative data were expressed as mean ± standard deviation, and qualitative data were expressed as frequency and percentage. Fisher’s exact test was used to investigate the statistical significance of the association between any two qualitative data sets. The Kruskal-Wallis test was used to detect the significance of differences in the levels of quantitative data when categorized according to grades of fibrosis and steatosis. A probability of an association or a difference less than 0.05 was considered significant.

## Results

Table 1 shows the demographic data of this study. Of the 50 patients, 27 (54%) were female and 23 (46%) were male. The mean age of the studied population was 47.72±8.31 years, with a minimum of 28 and a maximum of 64 years. The mean BMI was 28.44 ± 4.24, with most participants being either overweight or obese. The mean FibroScan result for fibrosis was 4.74 ± 1.02 kPa, while the mean result for steatosis was 282.88 ± 44.99. Most of our patients were housewives, and the second most common occupation was self-employment (28%). Fibrosis stage zero (F0) was the most frequent result, while steatosis stage three (S3) was most common in steatosis.

**Table 1:** Basic characteristics of the study population.

Variables		Mean± SD	Median (Min.-Max.)
Age		47.72±8.31	47 (28-64)
BMI		28.44±4.24	28.33 (19.23-36.1)
FibroScan results of fibrosis		4.74±1.02	4.55 (3-7.5)
FibroScan results of steatosis		282.88±44.99	284 (209-400)
		Frequency	Percent
Sex	Male	23	46.0
	Female	27	54.0
Occupation	Housewife	25	50.0
	Self employed	14	28.0
	Employee	10	20.0
	Retired	1	2.0
Fibrosis	0	42	84.0
	1	6	12.0
	2	2	4.0
	3	0	0
	4	0	0
Steatosis	0	9	18.0
	1	7	14.0
	2	13	26.0
	3	21	42.0
	Total	50	100.0

In Table 2, there was no significant association between fibrosis stage and patients’ sex, occupation, type of treatment for DM, hypertension, or the type of drugs used for hypertension, with p-values of (0.354, 0.209, 0.188, 0.239, 0.239) respectively.

**Table 2:** Association of some independent factors with liver fibrosis. * Fisher's Exact Test HTN: Hypertension.

Variables		Fibrosis stage			Total	P-value
		0	1	2		
Sex N (%)	Male	19	2	2	23	0.354
		45.2%	33.3%	100%	46%	
	Female	23	4	0	27	
		54.8%	66.7%	0%	54%	
Occupation N (%)	House wife	21	4	0	25	0.209
		50%	66.7%	0%	50%	
	Self employed	12	2	0	14	
		28.6%	33.3%	0%	28%	
	Employee	8	0	2	10	
		19%	0%	100%	20%	
	Retired	1	0	0	1	
		2.4%	0%	0%	2%	
Type of treatment N (%)	Insulin	9	2	1	12	0.188
		21.4%	33.3%	50%	24%	
	Oral antidiabetics	28	4	0	32	
		66.7%	66.7%	0%	64%	
	Insulin+ oral antidiabetics	5	0	1	6	
		11.9%	0%	50%	12%	
HTN N (%)	No	21	1	1	23	0.239
		50%	16.7%	50%	46%	
	Yes	21 (50%)	5 (83.3%)	1 (50%)	27	
		50%	83.3%	50%	54%	
HTN treatment N (%)	No	21 (50%)	1 (16.7%)	1 (50%)	23	0.239
		50%	16.7%	50%	46%	
	Yes	21	5	1	27	
		50%	83.3%	50%	54%	
Total		42	6	2	50%	

Table 3 shows that there was no significant association between the steatosis grade measured by FibroScan and the studied patients’ sex, occupation, type of diabetes treatment, hypertension, and the hypertension medication used, with p-values of (0.461, 0.917, 0.450, 0.316, 0.316).

**Table 3:** Association of some independent factors with steatosis. * Fisher's exact test.

		Steatosis				Total	P-value
		0	1	2	3		
Sex	Male	6	4	5	8	23	0.461
		66.7%	57.1%	38.5%	38.1%	46.0%	
	Female	3	3	8	13	27	
		33.3%	42.9%	61.5%	61.9%	54.0%	
Occupation	Housewife	3	3	7	12	25	0.917
		33.3%	42.9%	53.8%	57.1%	50.0%	
	Self employed	4	2	3	5	14	
		44.4%	28.6%	23.1%	23.8%	28.0%	
	Employee	2	2	3	3	10	
		22.2%	28.6%	23.1%	14.3%	20.0%	
	Retired	0	0	0	1	1	
		0.0%	0.0%	0.0%	4.8%	2.0%	
Type of treatment	Insulin injection	0	3	4	5	12	0.450
		0.0%	42.9%	30.8%	23.8%	24.0%	
	Oral Rx	8	4	7	13	32	
		88.9%	57.1%	53.8%	61.9%	64.0%	
	Insulin injection + Oral Rx	1	0	2	3	6	
		11.1%	0.0%	15.4%	14.3%	12.0%	
Hypertension (HT)	No	4	5	7	7	23	0.316
		44.4%	71.4%	53.8%	33.3%	46.0%	
	Yes	5	2	6	14	27	
		55.6%	28.6%	46.2%	66.7%	54.0%	
HT drugs	No	4	5	7	7	23	0.316
		44.4%	71.4%	53.8%	33.3%	46.0%	
	Yes	5	2	6	14	27	
		55.6%	28.6%	46.2%	66.7%	54.0%	
Total		9	7	13	21	50	
		100.0%	100.0%	100.0%	100.0%	100.0%	

When comparing the differences in age, duration of diabetes, BMI, systolic BP, diastolic BP, fasting blood sugar (FBS), AST, ALT, serum cholesterol, serum triglyceride, and HDL according to the stage of fibrosis measured by FibroScan, no significant statistical association was observed, with p-values of (0.241, 0.131, 1.000, 1.000, 0.855, 0.615, 0.287, 0.613, 1.000, 0.314, 0.613) as shown in Table 4.

**Table 4:** Differences in some basic characteristics according to the stage of fibrosis. *Kruskal Wallis test is used to determine the statistical significance. DM: Diabetes Mellitus; BP: Blood Pressure; FBS: Fasting Blood Sugar; AST: Aspartate Aminotransferase; ALT: Alanine Aminotransferase; HDL: High-Density Lipoprotein.

Variables	Fibrosis stage									P-values
	0			1			2			
	N	Mean	SD	N	Mean	SD	N	Mean	SD	
Age	42	46.98	8.11	6	53.83	8.61	2	45.00	7.07	0.241
Duration of DM (year)	42	7.22	5.65	6	9.33	6.31	2	17.50	3.53	0.131
BMI (kg/m²)	42	29.07	4.40	6	28.33	4.50	2	28.00	1.41	1.000
Systolic BP (mmHg)	42	127.02	14.44	6	126.33	18.99	2	125.00	21.21	1.000
Diastolic BP (mmHg)	42	85.12	11.07	6	80.83	10.20	2	80.00	14.14	0.855
FBS (mg/dL)	42	268.14	98.39	6	227.67	111.99	2	171.00	41.01	0.615
AST (U/L)	42	25.33	10.59	6	18.80	3.23	2	19.00	1.41	0.287
ALT (U/L)	42	25.78	17.21	6	14.81	3.39	2	19.00	8.48	0.613
Serum Cholesterol (mg/dL)	42	191.45	62.69	6	254.50	201.03	2	182.00	45.25	1.000
Serum Triglycerides (mg/dL)	42	218.01	126.66	6	205.50	151.68	2	217.00	38.18	0.314
Serum HDL (mg/dL)	42	42.83	12.02	6	38.75	14.42	2	38.00	2.82	0.613

However, diastolic BP, serum ALT, and serum HDL levels were the variables that showed statistically significant differences when compared according to the stages of steatosis measured by FibroScan, with p-values of (0.016, 0.048, 0.028), respectively. No significant association was found between steatosis grade and the patients’ age, duration of diabetes, BMI, systolic blood pressure, FBS, AST, serum cholesterol, and serum triglyceride level, with p-values of (0.487, 0.313, 0.266, 0.085, 0.639, 0.643, 0.988, 0.261), as shown in Table 5.

**Table 5:** Differences in some basic characteristics according to the grades of steatosis. *Kruskal Wallis test is used to determine the statistical significance. DM: Diabetes Mellitus; BP: Blood Pressure; FBS: Fasting Blood Sugar; AST: Aspartate Aminotransferase; ALT: Alanine Aminotransferase; HDL: High-Density Lipoprotein.

Variables	Steatosis grade													P-values
	0			1			2			3				
	N	Mean	^*^SD	N	Mean	SD	N	Mean	SD	N	Mean		SD	
Age	9	46.78	4.23	7	43.57	11.38	13	48.46	9.65	21	49.05		7.65	0.487
Duration of DM (year)	9	6.11	4.64	7	10.57	5.62	13	8.46	6.15	21	7.39		6.45	0.313
BMI (kg/m²)	9	26.56	4.33	7	27.01	5.15	13	28.69	3.11	21	30.52		4.26	0.266
Systolic BP (mmHg)	9	128.89	10.54	7	117.14	11.12	13	122.31	12.35	21	132.05		17.06	0.085
Diastolic BP (mmHg)	9	89.44	10.13	7	75.71	7.86	13	80.00	9.12	21	87.86		11.01	0.016
FBS (mg/dL)	9	265.67	109.17	7	295.71	112.76	13	241.92	81.46	21	255.43		105.12	0.639
AST (U/L)	9	24.41	11.53	7	21.18	7.93	13	24.26	10.51	21	25.31		10.19	0.643
ALT (U/L)	9	19.35	6.20	7	19.67	8.88	13	17.92	6.97	21	31.66		21.86	0.048
Serum Cholesterol (mg/dL)	9	187.24	54.72	7	190.85	62.79	13	194.15	75.00	21	208.90		115.65	0.988
Serum Triglycerides (mg/dL)	9	187.59	110.99	7	175.00	48.29	13	195.15	103.08	21	255.86		154.44	0.261
Serum HDL (mg/dL)	9	49.44	30.50	7	41.71	8.47	13	47.39	14.26	21		35.92	7.36	0.028

## Discussion

This study, conducted on 50 patients with T2DM using the FibroScan, showed that 23 patients were males and 27 were females. The mean liver stiffness measurement was 4.74, indicating stage 1 fibrosis, while the mean steatosis score was 282.88, indicative of stage 3 steatosis. This advanced steatosis score is attributed to the presence of diabetes mellitus and metabolic syndrome in most of the studied patients.

This study showed no significant association between patients’ age and the fibrosis score measured by the FibroScan, similar to the findings of Shrestha R et al. [26]

Additionally, this study assessed the association of the duration of diabetes, patients' BMI, blood pressure, serum ALT, AST, fasting blood sugar, serum cholesterol, triglyceride levels, and HDL cholesterol with the fibrosis score. No significant associations were found, which contrasts with the results from a study by Lai LL et al., which identified significant correlations between liver stiffness and BMI, waist circumference, fasting blood sugar level, and liver enzymes. However, triglyceride levels, high-density lipoprotein levels, and the duration of diabetes mellitus did not significantly correlate with liver stiffness [27].

This study differs from a study done in Japan (2018) by Tada T et al. that showed a significant association between gender and liver stiffness and the severity of stiffness (indicative of fibrosis), showing 55% of males and 39% of females with increased liver stiffness and about 14% of males and 11% of females with severe fibrosis. Our study, however, found no significant association between patients' sex and the degree of fibrosis [28].

In our study, serum ALT levels were found to be significantly associated (p-value=0.048) with the stage of steatosis measured by FibroScan, while serum AST levels were not significantly associated (p-value=0.643), aligning with the results of a study by Ma Q et al. [29]. This finding could be explained by the fact that AST elevation is not specific to liver disease, while ALT levels are indicative of liver injury.

Furthermore, this study found a significant association between diastolic blood pressure and the steatosis grade, consistent with findings from a study conducted in China in 2016 by Qian LY et al. This correlation could be explained by the fact that NAFLD and blood pressure are components of metabolic syndrome [30].

In a 2019 study conducted at a hospital in Ohio, USA, it was found that all patients with NAFLD had decreased HDL levels compared to healthy controls, and the HDL level was negatively correlated with the steatosis score measured by FibroScan. These results were similar to those reached in our study [27]. This could again be explained by the fact that serum HDL is a component of metabolic syndrome and contributes to insulin resistance, which is the fundamental mechanism for the development of NAFLD.

In our study, we found no significant association between BMI and steatosis score, although the mean BMI value was slightly higher in our studied patients. This contrasts with the findings of Alsabaani AA et al., where BMI was considered an independent risk factor for the accumulation of fat in the liver, potentially due to the large sample size included in their study [8].

The limitations of this study can be summarized by its single-center nature and the small sample size, attributed to the limited availability of the FibroScan machine.

## Conclusions

Some risk factors associated with diabetes, such as dyslipidemia, elevated liver enzymes, and blood pressure, are highly associated with the development of steatosis rather than fibrosis.

This study concluded that NAFLD is common in patients with T2DM, and most diabetic patients with NAFLD present with an advanced grade of steatosis at the time of diagnosis. Despite having normal liver function and a low grade of associated fibrosis, these patients carry a favorable prognosis at this stage. This form of steatosis can be reversible, and with appropriate interventions, it is possible to halt and reverse the progression to steatohepatitis and advanced hepatic fibrosis.
